# Supplementary material for: Associations between high blood pressure and DNA methylation
Source: PLoS One. 2020 Jan 30;15(1):e0227728. doi: 10.1371/journal.pone.0227728 (PMC6991984; doi:10.1371/journal.pone.0227728)
Supplement: Attachment — Submitted filename: Rebuttal letter.docx [file pone.0227728.s012.docx]

**Manuscript:** **Associations Between High Blood Pressure and DNA Methylation**

**Reviewer #1:**

**Comment 1:** Line 187. Differentially methylated region analysis. I know the authors found four regions of overlap, but I was wondering if the authors looked to see if the other discrepant regions happened to fall in similar pathways?

**Response:** Now, we have performed the pathway analyses and revised the manuscript accordingly.

**Results:**

“*We performed pathway analyses for the genes mapped for DMRs of SBP and DBP analyses conducted for South Asian and European groups. For SBP analyses conducted for Europeans and DBP analyses conducted for South Asians, we identified pathways enriched with FDR q-value<0.05. The identified pathways for SBP analyses conducted for Europeans were NOTCH2 intracellular domain regulates transcription and NOTCH4 intracellular domain regulates transcription. The identified pathway for DBP analyses conducted for South Asians was insulin-like growth factor-2 mRNA binding proteins (IGF2BPs/IMPs/VICKZs) bind RNA.”*(pages:11-12; lines: 217-224)

**Methods:**

*“Pathway analyses were carried out using the reactome (*[*https://reactome.org/*](https://reactome.org/)*) to test the genes mapped for DMRs for enrichment of certain biological pathways.”*(page:22; lines: 452-453)

**Comment 2:** Line 216. This is more of a formatting issue. "Known genetic variants in DMRs" is on the same line as Figure 4 legend.

**Response:** We have corrected the formatting issue. Please see line 226 on page 12.

**Comment 3:** Line 370. When were the traits measured? Were they also at baseline?

**Response:** Yes, please see:

*“Blood pressure was measured on one occasion (the average of 2 consecutive readings) in the baseline research clinic as described previously (46).” (pages 17-18; lines: 366-367)*
